# Food waste awareness among Italian university students: results of an online survey

**DOI:** 10.3389/fnut.2024.1401581

**Published:** 2024-08-05

**Authors:** Federica Catalano, Velia Cassano, Arturo Pujia, Angela Sciacqua, Marta Letizia Hribal

**Affiliations:** ^1^Department of Medical and Surgical Sciences, University Magna Græcia of Catanzaro, Catanzaro, Italy; ^2^Research Center for the Prevention and Treatment of Metabolic Diseases, University Magna Græcia of Catanzaro, Catanzaro, Italy

**Keywords:** gender related differences, education, waste management and disposal, university student behavior, expiration date monitoring

## Abstract

**Introduction:**

Food waste (FW) represents a significant social and environmental problem, with 1.3 billion tons of food wasted yearly worldwide. Even if consumers are increasingly aware of the phenomenon, it remains relevant, and understanding the behaviors of specific target audience segments appears instrumental to the planning of effective interventions. To this end, we designed an observational study to investigate, throughout an online questionnaire, FW-related habits of university students in a Southern Italian region.

**Methods:**

A representative sample of 431 students from the University of Catanzaro Magna Graecia completed an online survey aimed at investigate FW related behaviors. A descriptive analysis was performed on the whole cohort, and a formal statistical analysis was carried out after excluding responders who had not correctly followed the survey instructions (*n* = 85). Differences were assessed by chi square (*χ*^2^) tests. A food wasting score was generated, and differences in the score values were analyzed by Student *T*-test. Linear and multiple regression analyses were performed to identify factors contributing to the score.

**Results:**

Overall, the results of our survey show a high prevalence of virtuous behaviors in the food purchasing phase; while, at home, less than 50% of respondents apply easy-to-implement waste prevention rules. The statistical analysis showed that the major determinants of FW were: no direct involvement in grocery shopping and male gender. Indeed, even if we observed several significant differences comparing subgroups based on established or putative determinants of FW behaviors, none survived matching for group size, gender and relevant food managing (shopping, storing, cooking) habits. The only exception was the more appropriate handling of “use by” products by respondents who received structured nutrition teaching, as opposed to students whose academic courses do not include this subject (adjusted *p* = 0.008).

**Conclusion:**

Our data suggest that young adults are trying to implement strategies to reduce FW, even if there is room for improvement, particularly in the storage phase. Extending nutrition education to all university programs may be helpful in reaching this goal.

## 1 Introduction

Food waste (FW) represents a significant social and environmental problem; it is defined as all comestible materials within the food reserve that are intended for human ingestion but are not consumed (1). FW not only results in wasted resources and avoidable environmental impact but has also an important cost for human health since wasted food represent nutrients and energy that could have provided nutritional benefits (2).

Every year, 1.3 billion tons of food are lost or wasted worldwide (3); the amount of FW is, however, different in every country and region. For example, consumers from Europe and North America waste a larger amount of food than consumers in sub-Saharan Africa and South/Southeast Asia (95–115 kg *per capita* per year vs. 6–11 kg *per capita* per year, respectively). It has been estimated that the total amount of waste generated by consumers in developed countries every year (222 billion kg) approximately equals the total amount of annual food production in sub-Saharan Africa (230 billion kg) (2). If we focus on Italy, the Southern Italian Regions present lower incidence of FW than Central or Northern regions (4).

FW occurs mainly at the later stages of the food supply chain, such as the retail and consumption phases. According to the Food Waste Index Report 2024 of the United Nations Environment Programme (UNEP), in 2022, 12% of the total waste was generated by retail vendors, 28% by food services, while 60% was due to households (5). Interestingly, it has been suggested that consumers also indirectly cause FW because the supply chain assumes that they demand perfect cosmetic quality related to the shape, size, and appearance of food products, which leads to waste in the primary production stage of the food chain (6).

In recent years, consumers are becoming increasingly aware of their role in preventing FW; however, despite the implementation of national programs aimed at addressing it, including the so-called Good Samaritan Law (155/2003) in Italy (7), the phenomenon remains relevant.

The European Union (EU) Platform on Food Losses and Food Waste has underlined the importance of understanding the behaviors and motivations of specific target audience segments to gain insights into obstacles to behavioral changes and plan more effective interventions (8).

Young adults, such as those attending university courses, represent an ideal population since they are usually more environmentally conscious than older generations, they can be more easily reached by intervention programs, and they are more likely to adopt behavioral changes. Up to 80% of the literature in the field of food-related habits is indeed based on students cohorts, according to a recent review (9); among these, there are studies carried out in Italy (10–15), particularly in the Southern regions of the country (11, 13, 15), as well as studies specifically addressing FW-related behaviors (10, 16, 17). However, to the best of our knowledge, the only studies directly addressing FW in an Italian cohort date to almost a decade ago and examined data from comparatively small (*n* = 180 and *n* = 233, respectively) groups of students living in Lazio, a Central Italian region (10, 12). We thus designed this observational, pilot study to investigate FW-related habits of students attending the University of Catanzaro Magna Graecia in Calabria, a Southern Italian region. While the mediterranean area has a solid tradition of healthy eating and a strongly rooted food culture (18), in more recent years, North–South and older adults-younger subjects decreasing gradients of nutrition literacy have been reported (19); our study will thus also contribute to this debate.

## 2 Methods

### 2.1 Questionnaire and survey methodology

A sample of *n* = 431 students enrolled in academic programs at the University of Catanzaro Magna Graecia, completed an online survey between November 22^nd^ 2022, and February 7^th^, 2023, aimed to investigate FW-related behaviors (Supplementary Figure S1). Students were invited to participate in the survey during regular frontal lessons on subjects related as well as unrelated to nutrition issues; participants were also asked to disseminate the questionnaire among their friends and peers (snowball sampling technique). Students from all academic programs offered by the University of Catanzaro Magna Graecia participated to the survey. A short statement explaining the survey purpose, the planned use of the data, reporting the eligibility criteria (i.e., to be 18 years or older and enrolled at the University of Catanzaro Magna Graecia), as well as stating that the data were anonymous and that the completion of the study would indicate consent, was included at the beginning of the survey. Each user could fill in the questionnaire only once. E-mail addresses were not recorded and were not visible to the study investigators to ensure anonymity. The Magna Graecia University Ethics Committee (Comitato Etico Azienda Ospedaliera “Mater Domini”) assessed the questionnaire and concluded that formal approval was not necessary because answers were anonymous and non-sensitive data only were collected.

The questionnaire included 23 one-option or multiple choices questions structured in 6 different sections. A translation of the Italian version, which was employed in the study, is available as Supplementary material. All questions have been developed by the study investigators since none of the questionnaires employed in previous studies were developed for our target population. Specifically, the survey was developed by one of the senior authors (MLH), and subsequently amended by another senior author (AP). The survey was then taken by the two youngest authors (FC and VC) who suggested a couple of improvements, and by a small group (*n* = 15) of students enrolled in the third final year of the dietistics degree course whose answers were not included in the final cohort (Supplementary Figure S1). The initial section (A) covered sociodemographic information such as age, gender, academic formation and living situation. Section B contained questions regarding grocery shopping, food storage and cooking habits; section C included a set of questions aimed at evaluating behaviors during the shopping phase; section D questions were devoted to the food storage phase; while section E questions were related to the meal preparation and handling stages. Finally, section F comprised questions related to waste and recycling. Three questions (Q7, Q13, and Q15) offered an “open answer” option; however, given the small number of individuals choosing this option, their responses were merged with the more similar fixed one for analysis purposes. The only mandatory questions were those in section B; therefore, the total number of responses was slightly different for some questions, both in the whole population and in the sample retained after omitting the respondents who did not follow the questionnaire instructions. The time required to complete the questionnaire ranged from 2 to 10 min (medium time: 4 min).

### 2.2 Statistical analysis

Data were analyzed using descriptive analysis and are expressed as relative frequencies or as mean ± standard deviation (SD), as appropriate. A formal statistical comparison was carried out after excluding respondents who answered questions that they were supposed to skip according to questionnaire instructions (i.e., Q8–11 with D or E for Q5, Q12–15 with C, D or E for Q6, Q16–19 with D, E or F for Q7). The retained sample included 346 students (Supplementary Figure S1). There were no significant differences in the characteristics of the excluded subjects for Q1–4. In addition, to simplify the interpretation of the results, we coded the responses to Q3 as follows: Q3.1 students enrolled in a biomedical area program vs. students enrolled in a program from a non-biomedical field; Q3.2: students enrolled in a program comprising at least one nutrition course vs. students enrolled in a program that does not include structured nutrition teaching. We also merged the responses to Q4 as follows: Q4.1 students living with their family of origin (old living situation) vs. students living in a new household (alone, spouse/partner with or without children, other students, etc.); Q4.2 students living with either their family of origin or their new family (spouse/partner and children) vs. students in other living situations (alone, spouse/partner without children, other students). Differences between groups were assessed by chi square (χ2) tests. A food wasting score was generated assigning 0 to 3 points for responses to questions Q8–19. Differences in the score values were analyzed by Student *T*-test. Linear and multiple regression analyses were performed to identify factors contributing to the score (Supplementary Figure S1). A *p*-value < 0.05 was considered statistically significant in all analyses. All analyses were performed using Jamovi 2.3.21 software version.

## 3 Results

**Figure 1:**
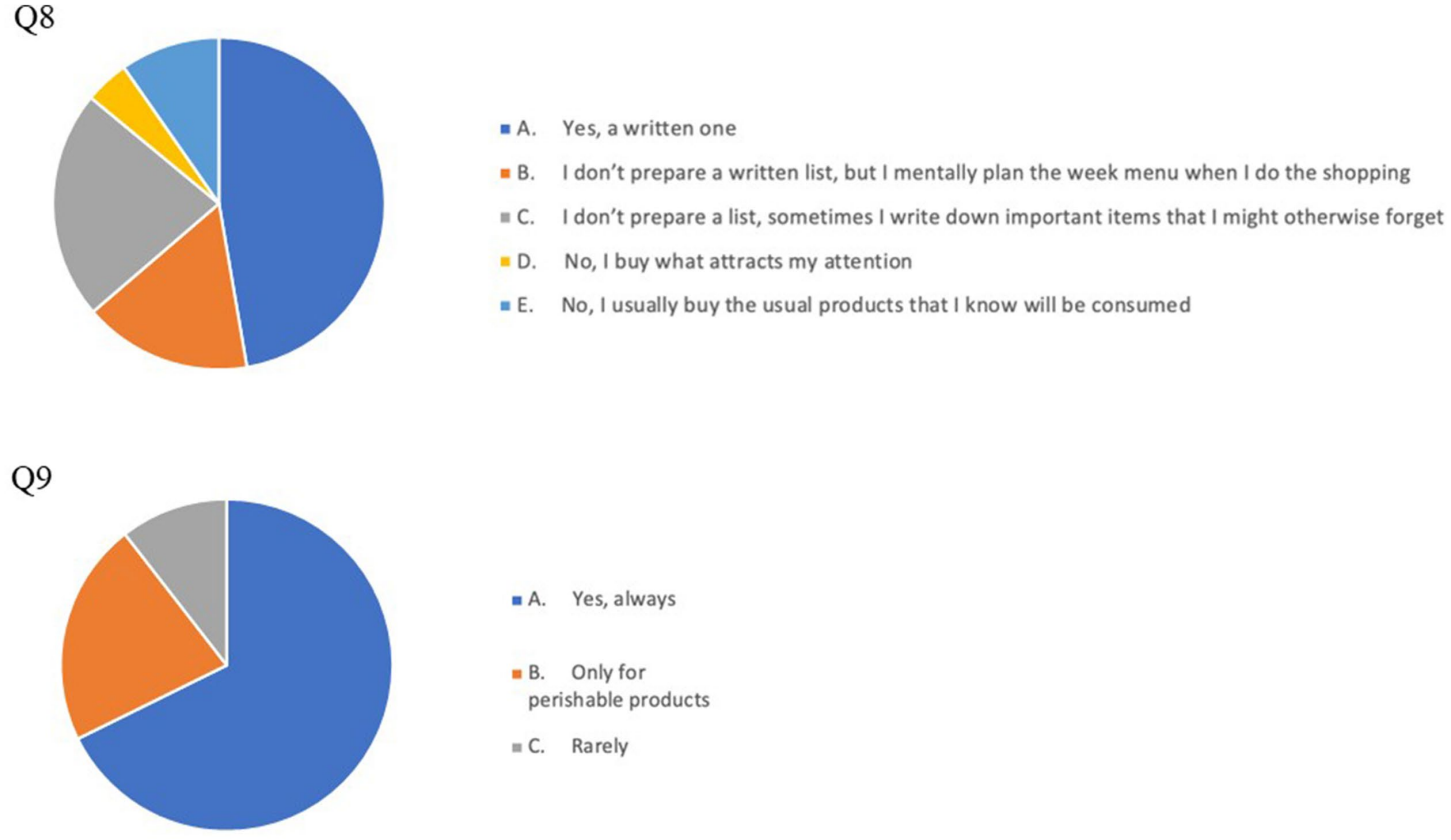
Pie charts of answers to Q8 (upper chart): Grocery shopping planning and Q9 (lower chart): Checking expiration dates when grocery shopping answers.

**Figure 2:**
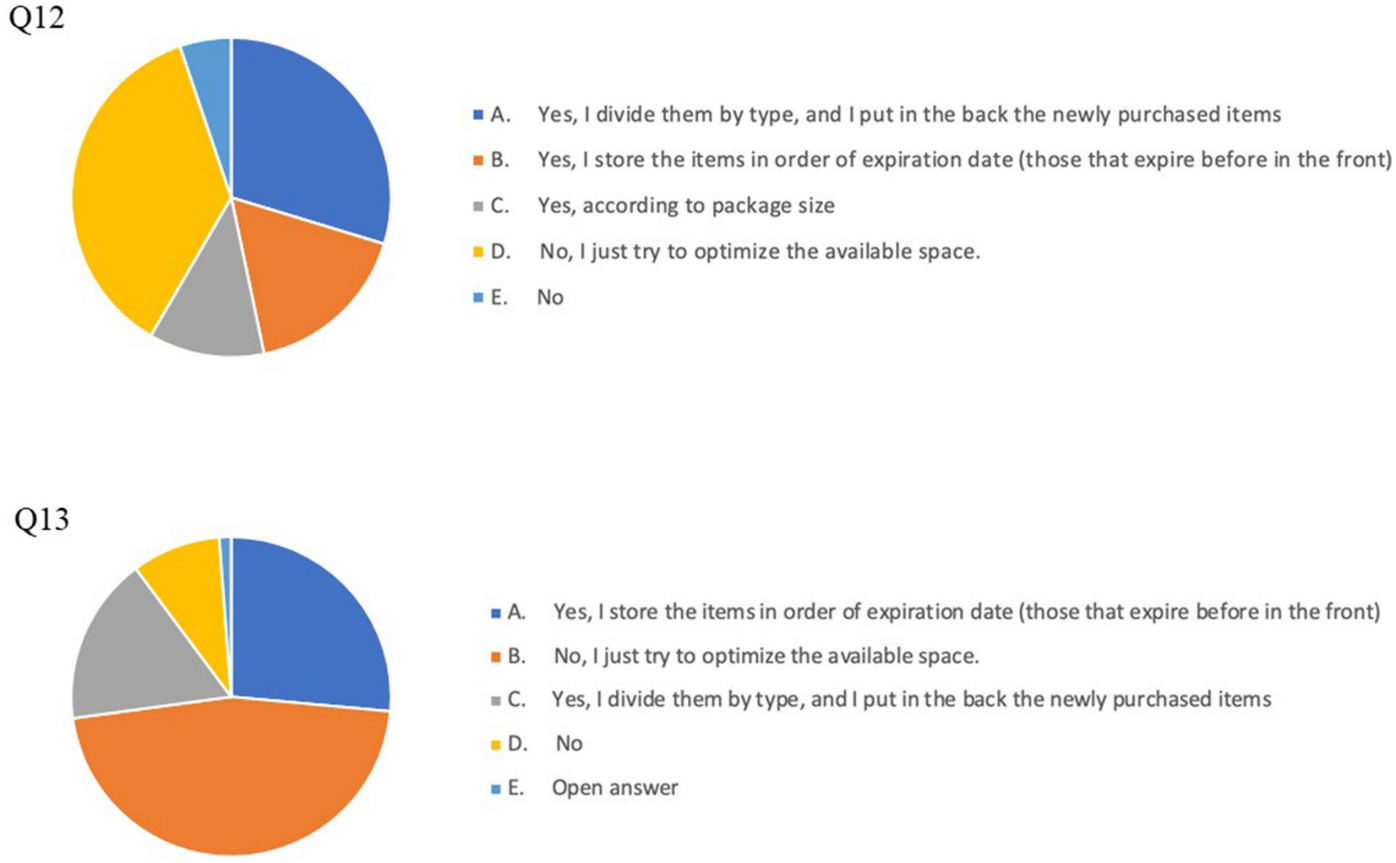
Pie charts of answers to Q12 (upper chart): Storage criteria in the cupboard and Q13 (lower chart): Storage criteria in the refrigerator.

**Figure 3:**
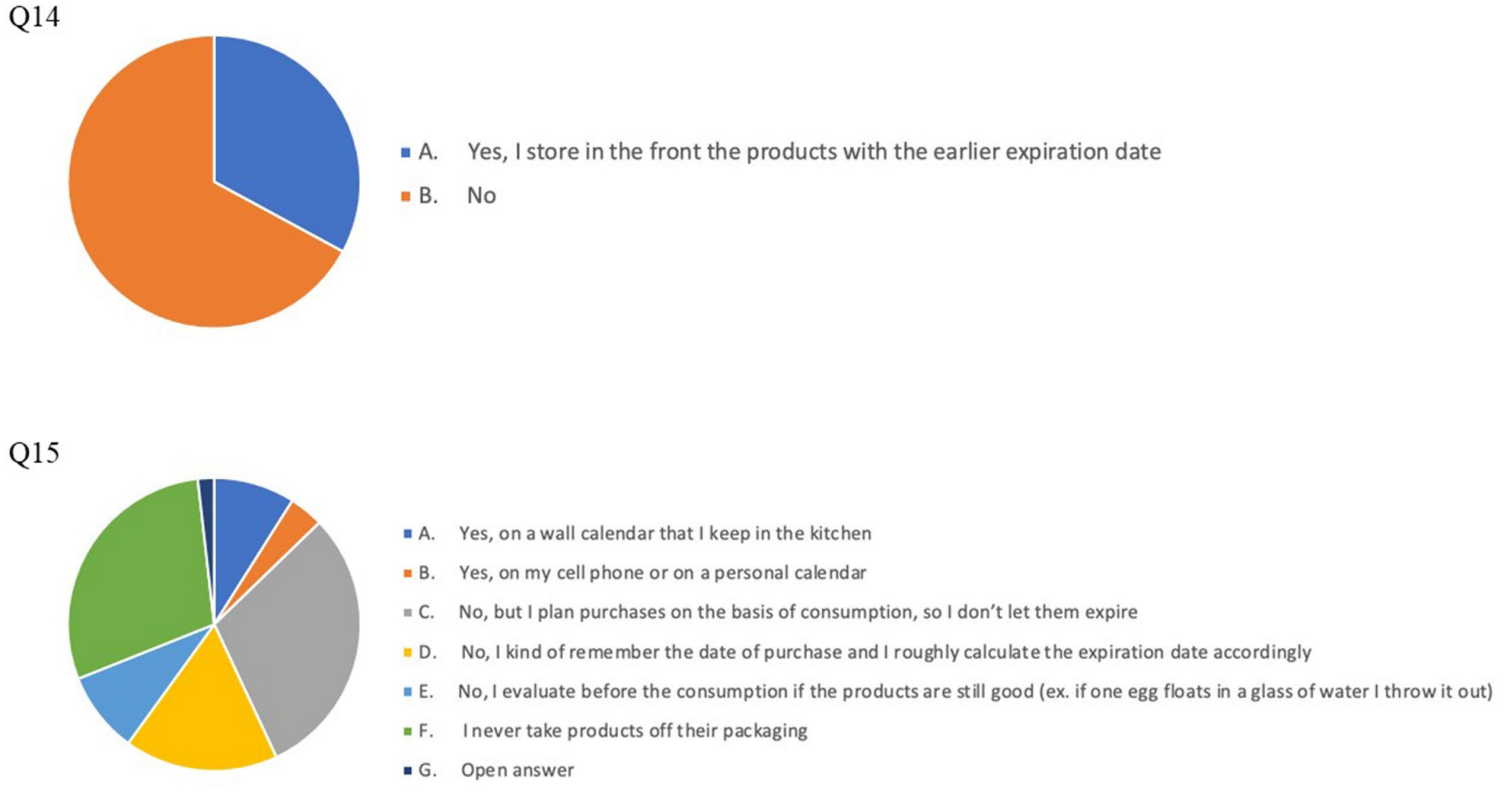
Pie charts of answers to Q14 (upper chart): Storage criteria in the freezer and Q15 (lower chart): Expiration date recording.

The results of our survey suggest a good prevalence of FW -reducing habits in our cohort: particularly, 252 respondents (67.7% of the whole cohort) always checked expiration dates and 237 (63.7%) declared to use a written list or a weekly menu, while grocery shopping (Figure 1). By contrast, a suboptimal percentage of the respondents stored food products according to their expiration dates or to any criterion aimed at reducing the risk of letting products go past their expiration date (Q12: *n* = 187, 46.7%; Q13: *n* = 174, 43.5%; Q14: *n* = 131, 32.9%) and a surprisingly high number of individuals (*n* = 189, 47.3%) relied only on their memory and ability to carefully plan food purchases and did not register expiration dates when removing products (e.g., eggs) from their original packaging (Figures 2, 3).

The mean age of the 431 respondents was 22.4 ± 3.34 (range 18–47); as expected, the majority of the sample (69.4%, *n* = 298) was in the 20–23 years old (y/o) range, with only 34 (7.9%) younger individuals (18 y/o, *n* = 4: 19 y/o: *n* = 30); 69 (16%) subjects were in the 24–26 y/o range, and 29 (6.6%) were older (27 y/o: *n* = 4; 28 y/o: *n* = 6; 29 y/o: *n* = 4; 30 y/o: *n* = 1; 31 y/o: *n* = 3; 32 y/o: *n* = 1; 33 y/o: *n* = 2; 34 y/o: *n* = 2; 35 y/o: *n* = 1; 37 y/o: *n* = 2; 41 y/o: *n* = 1; 43 y/o: *n* = 1; 47 y/o: *n* = 1). The distribution of the responses to Q2-23 is summarized in Figure 1 and Supplementary Figure S2. The female gender was predominant (268 F vs. 161 M; 62.5% F vs. 37.5% M; Supplementary Figure S2A), reflecting not only the gender distribution of the students enrolled at the University of Catanzaro Magna Graecia, but also the higher willingness of girls and women to respond to surveys, observed across studies (15, 20, 21). The majority of the respondents still lived at home with their parents/family of origin (*n* = 255; 59.2%), 34 (7.9%) lived alone or with a partner with or without children; 142 (33%) lived with roommates; there were no students living in residence halls or other residential communities (Supplementary Figure S2B); 63.1% (*n* = 272) of the entire group personally shopped for groceries (Supplementary Figure S2B), and 91.3% (*n* = 394) was, at least occasionally, involved in meal preparation (Supplementary Figure S2C).

Notably, the vast majority of the whole sample (93.3%, *n* = 401) reported following local separate waste collection rules; even if this response is probably influenced by a social desirability bias, this observation is in keeping with the data on waste disposal in the Catanzaro urban area (Supplementary Figure S2D) (22). It is also worth noting that 61.5% (*n* = 260) of the respondents reported disposing mainly of metal/plastic waste, while the least common response to the question about the type of waste produced in greater amounts was glass (*n* = 3, 0.7%). In addition, “vegetables and fruits gone bad” was the most common response to Q23 (*n* = 112; 26.1%) followed by “meal leftovers” (*n* = 77; 17.9%), and “expired products, not consumed (*n* = 74; 17.2%; Supplementary Figure S2E).

When we performed a formal statistical analysis on the retained cohort (*n* = 346) obtained after omitting the respondent who did not correctly follow the survey instructions, as detailed in paragraph 2.2, we found significant differences for section B questions (Q5–7) with female students more frequently taking care of food storage and cooking; students enrolled in biomedical area programs more frequently in charge of grocery shopping and cooking; and students living in a new household as well as those living in a non-family household more frequently involved in all three activities (grocery shopping/food storage/cooking). Across the entire sample, 69.9 and 65.6%, respectively, of the respondents who did their own grocery shopping at least once a month took care of food storage at least in part and of cooking their meals, and 62.9% of those who took care of food storage were also responsible for meal preparation. In addition, we observed that male students more frequently did their shopping with a written list, while female students more frequently stored the food items in the refrigerator according to waste-reducing criteria and were less likely to throw away vegetables and fruits when they are no longer fresh. These differences were, however, no longer significant when we matched M/F groups for the number of individuals and section B responses (Tables 1–3).

**Table 1:** Results of *χ*^2^ analysis for Q5, Q6 and Q7.

Q5. Grocery shopping	A (Always)	B (Usually)	C (Sometimes)	D (Rarely)	E (Never)	*χ*^2^	*p*
Biomedical/other area	Obs:133/5 Exp:128.43/9.57	Obs:101/5 Exp:98.65/7.35	Obs:59/8 Exp:62.35/4.65	Obs:19/6 Exp:23.27/1.73	Obs:10/0 Exp:9.31/0.69	17.8	0.001
Biomedical/other area no outliers	Obs:122/5 Exp:117.87/9.13	Obs:93/5 Exp:90.96/7.04	Obs:53/7 Exp:55.69/4.31	Obs:19/6 Exp:23.20/1.80	Obs:10/0 Exp:9.28/0.72	15.8	0.003
Family/ other living situation	Obs:23/115 Exp:77/61	Obs:73/33 Exp:59.1/46.9	Obs:63/4 Exp:37.4/29.6	Obs:24/1 Exp:13.9/11.1	Obs:10/0 Exp:5.58/4.42	157	<0.001
Family/other living situation no outliers	Obs:20/107 Exp:70.6/56.4	Obs:67/31 Exp:54.5/43.5	Obs:57/3 Exp:33.4/26.6	Obs:24/1 Exp:13.9/11.1	Obs:10/0 Exp:5.56/4.44	150	<0.001
New/old living situations	Obs:119/19 Exp: 65.8/72.2	Obs:36/70 Exp: 50.5/55.5	Obs:36/70 Exp: 50.5/55.5	Obs:36/70 Exp:50.5/55.5	Obs:0/10 Exp:4.77/5.23	150	<0.001
New/old living situations no outliers	Obs:108/19 Exp:58.7/68.3	Obs:31/57 Exp:45.3/52.7	Obs:8/52 Exp:27.8/32.3	Obs:1/24 Exp:11.6/13.4	Obs:0/10 Exp:4.63/5.38	138	<0.001
Q6. Food storage	A (Always)	B (Usually)	C (Partly)	D (Never)	E (never Q5 and Q6)	*χ*^2^	*p*
M/F	Obs:57/128 Exp:62.9/122.1	Obs:47/91 Exp:46.9/91.1	Obs:5/2 Exp:2.38/4.62	Obs:3/4 Exp:2.38/4.62	Obs:5/2 Exp:2.38/4.62	9.82	0.044
M/F no outliers	Obs:53/116 Exp:58.3/110.7	Obs:44/86 Exp:44.8/85.2	Obs:5/2 Exp:2.41/4.59	Obs:3/3 Exp:2.07/3.93	Obs:5/2 Exp:2.41/4.59	9.85	0.043
Family/other living situation	Obs:69/116 Exp:103.2/81.8	Obs:103/37 Exp:78.1/61.9	Obs:7/0 Exp:3.90/3.10	Obs:7/0 Exp:3.90/3.10	Obs:7/0 Exp:3.90/3.10	60.2	<0.001
Family/other living situation no outliers	Obs:64/105 Exp:94/75	Obs:94/37 Exp:72.9/58.1	Obs:7/0 Exp:3.89/3.11	Obs:6/0 Exp:3.34/2.66	Obs:7/0 Exp:3.89/3.11	51.3	<0.001
New/old living situations	Obs:124/61 Exp:88.2/96.8	Obs:40/100 Exp:66.8/73.2	Obs:0/7 Exp:3.34/3.66	Obs:1/6 Exp:3.34/3.66	Obs:0/7 Exp:3.34/3.66	64.1	<0.001
New/old living situations no outliers	Obs:109/60 Exp:78.2/90.8	Obs:39/92 Exp:60.6/70.4	Obs:0/7 Exp:3.24/3.76	Obs:0/6 Exp:2.77/3.23	Obs:0/7 Exp:3.24/3.76	54.2	<0.001
Q7. Cooking	A (Yes, for self)	B (yes for household)	C (Sometimes)	D (Never)	E (Rarely)	*χ*^2^	*p*
M/F	Obs:39/62 Exp:34.4/66.6	Obs:21/76 Exp:33/64	Obs:40/75 Exp:39.1/75.9	Obs:8/5 Exp:4.42/8.58	Obs:7/6 Exp:4.42/8.58	19.7	0.020
M/F no outliers	Obs:35/59 Exp:32.4/61.6	Obs:19/68 Exp:30/57	Obs:41/70 Exp:38.3/72.7	Obs:8/5 Exp:4.48/8.52	Obs:7/7 Exp:4.83/9.17	12.5	0.014
Biomedical/other area	Obs:99/2 Exp:93.99/7.01	Obs:85/12 Exp:90.27/6.73	Obs:113/7 Exp:111.68/8.32	Obs:14/0 Exp:13.03/0.97	Obs:11/3 Exp:13.03/0.97	14.1	0.007
Biomedical/other area no outliers	Obs:92/2 Exp:87.24/6.76	Obs:75/12 Exp:80.75/6.25	Obs:105/6 Exp:103.02/7.98	Obs:14/0 Exp:12.99/1.01	Obs:11/3 Exp:12.99/1.01	15.2	0.004
Family/other living situation	Obs:25/76 Exp:56.3/44.7	Obs:57/40 Exp:54.1/42.9	Obs:84/36 Exp:66.9/53.1	Obs:14/0 Exp:7.81/6.19	Obs:13/1 Exp:7.81/6.19	68.8	<0.001
Family/other living situation no outliers	Obs:23/71 Exp:52.3/41.7	Obs:50/37 Exp:48.4/38.6	Obs:78/33 Exp:61.7/49.3	Obs:14/0 Exp:7.79/6.21	Obs:13/1 Exp:7.79/6.21	65.8	<0.001
New/old living situations	Obs:77/24 Exp:48.2/52.8	Obs:47/50 Exp:46.7/50.7	Obs:39/77 Exp:55.3/60.7	Obs:0/14 Exp:6.68/7.32	Obs:0/13 Exp:6.20/6.80	71.8	<0.001
New/old living situations no outliers	Obs:72/22 Exp:43.5/50.5	Obs:40/47 Exp:40.2/46.8	Obs:35/76 Exp:51.3/59.7	Obs:0/14 Exp:6.47/7.53	Obs:1/13 Exp:6.47/7.53	65.2	<0.001

Obs, observed; Exp, expected; M, male; F, female; *χ*^2^, chi-square; *p*, *p*-value.

**Table 2:** Results of *χ*^2^ analysis for Q8.

Q8. Grocery shopping planning	A (Yes, written)	B (Weekly menu)	C (Note important items)	D (Impulsive shopping)	E (Routinary shopping)	*χ*^2^	*p*
M/F	Obs:47/97 Exp:44.7/99.3	Obs:18/29 Exp:14.6/32.4	Obs:12/56 Exp:21.1/46.9	Obs:3/7 Exp:3.10/6.90	Obs:10/11 Exp:6.52/14.48	9.73	0.045
M/F no outliers	Obs:44/85 Exp:40.74/88.26	Obs:16/27 Exp:13.58/29.42	Obs:11/54 Exp:20.53/44.47	Obs:3/5 Exp:2.53/5.47	Obs:10/11 Exp:6.63/14.37	10.11	0.039

Obs, observed; Exp, expected; M, male; F, female; *χ*^2^, chi-square; *p*, *p*-value.

**Table 3:** Results of *χ*^2^ analysis for Q13.

Q13. Storage criteria in the refrigerator	A (Expiration date)	B (Space optimization)	C (Type)	D (None)	*χ*^2^	*p*
M/F	Obs:27/61 Exp:27.8/60.2	Obs:54/91 Exp:45.9/99.1	Obs:9/47 Exp:17.7/38.3	Obs:10/17 Exp:8.54/18.46	8.78	0.032
M/F no outliers	Obs:24/56 Exp:25.5/54.5	Obs:52/79 Exp:41.7/89.3	Obs:8/47 Exp:17.5/37.5	Obs:9/17 Exp:8.28/17.72	11.5	0.009

Obs, observed; Exp, expected; M, male; F, female; *χ*^2^, chi-square; *p*, *p*-value.

In addition, respondents living in a family type household as compared with students living in a different type of household as well as students living with their family of origin (old living situation) as compared with students living in a novel household more frequently consumed meals leftover at later meals. However, they also reported slightly more frequently being unable to avoid them; this results in a significant difference in the type of FW, with meal leftovers being the most frequent in these two groups of individuals. All these differences were no longer significant when we matched family/other living situations and new/old living situations groups for group size and Section B responses (Table 4).

**Table 4:** Results of *χ*^2^ analysis for Q16 and Q17.

Q16. Fruits and vegetable no longer fresh	A (Throw them away)	B (Save good parts)	C (Cook them)	*χ*^2^	*p*
M/F	Obs: 25/31 Exp: 17.6/38.4	Obs: 66/161 Exp: 71.5/115	Obs:7/21 Exp:8.82/19.18	5.65	0.050
M/F no outliers	Obs:24/28 Exp:16.67/35.33	Obs:62/148 Exp:67.32/142.68	Obs:6/19 Exp:8.01/16.99	6.11	0.047
Family/ other living situation	Obs:20/36 Exp:28.90/27.10	Obs:128/100 Exp:117.65/110.35	Obs:13/15 Exp:14.45/13.55	7.84	0.020
Family/other living situation no outliers	Obs:17/35 Exp:26.63/25.37	Obs:118/92 Exp:107.56/102.44	Obs:12/13 Exp:12.80/12.20	9.32	0.009
New/old living situations	Ob:38/18 Exp:29.26/26.74	Ob:109/119 Exp:119.12/108.88	Ob:16/12 Exp:14.63/13.37	7.54	0.023
New/old living situations no outliers	Obs:36/16 Exp:26.45/25.55	Obs:97/113 Exp:106.83/103.17	Obs:13/12 Exp:12.72/12.28	8.87	0.012
Q17. Meal leftovers	A (Throw them away)	B (Consume later)	C (No leftovers)	*χ*^2^	p
Family/other living situation	Obs:11/10 Exp:10.83/10.17	Obs:90/62 Exp:78.42/73.58	Obs:61/80 Exp:72.75/68.25	7.45	0.024
Family/other living situation no outliers	Obs:7/10 Exp:8.71/8.29	Obs:85/58 Exp:73.2/69.8	Obs:56/73 Exp:66.1/62.9	7.70	0.021
Family/other living situation matched for age and gender	Obs:8/9 Exp:7.61/9.39	Obs:58/48 Exp:47.5/58.5	Obs:37/70 Exp:47.9/59.1	8.77	0.012
New/old living situations	Obs:12/9 Exp:10.97/10.03	Obs:67/85 Exp:79.39/72.61	Obs:85/56 Exp:73.64/67.36	7.92	0.019
New/old living situations no outliers	Obs:10/7 Exp:8.65/8.35	Obs:62/81 Exp:72.74/70.26	Obs:75/54 Exp:65.62/63.38	6.39	0.041

Obs, observed; Exp, expected; M, male; F, female; *χ*^2^, chi-square; *p*, *p*-value.

In addition, while 97.6% of the respondents affirmed knowing the difference between “use by” and “best before” labeling (66.7% “Yes for sure,” 30.9% “Yes, I think so”; Supplementary Figure S3A); 27.8 and 13.4%, respectively, gave incorrect answers when asked how they handled expired products in the two categories (Q18 and Q19, Supplementary Figure S3B). Interestingly, students enrolled in a program comprising at least one nutrition course gave the correct answers to Q18 significantly more frequently. This difference remained significant after matching the groups for group size and gender and also when the analysis was restricted to students enrolled in programs in the biomedical area and further matched for group size and gender (Table 5).

**Table 5:** Results of *χ*^2^ analysis for Q18.

Q18. Expired “use by” products	A (Taste and use)	B (Use good parts)	C (Throw away)	D (Cook, with caution)	E (rarely happens)	*χ*^2^	*p*
Nutrition course(s)/no nutrition course	Obs:23/46 Exp:27.9/41.1	Obs:6/9 Exp:6.07/8.93	Obs:76/72 Exp:59.9/88.1	Obs: 0/3 Exp:1.25/1.75	Obs:22/57 Exp: 32/47	16	0.003
Nutrition course(s)/no nutrition course no outliers	Obs:23/40 Exp:26.16/36.84	Obs:4/8 Exp:4.98/7.02	Obs:73/85 Exp:57.3/80.7	Obs:0/3 Exp:1.25/1.75	Obs:20/53 Exp:30.31/42.69	16.47	0.002
Nutrition course(s)/no nutrition courses matched for gender	Obs:23/21 Exp:25.9/18.1	Obs:6/1 Exp:4.12/2.88	Obs:76/37 Exp:66.4/46.6	Obs:0/1 Exp:0.59/0.41	Obs:22/29 Exp:30/21	12.8	0.012
Nutrition course(s)/no nutrition course (biomedical area cohort)	Obs:23/38 Exp:26.4/34.6	Obs:6/8 Exp:6.07/7.93	Obs:76/64 Exp:60.7/79.3	Obs: 0/2 Exp:0.86/1.13	Obs:22/54 Exp:32.9/43.1	15.6	0.004
Nutrition course(s)/no nutrition course (biomedical area cohort) matched for gender	Obs: 23/24 Exp:23/24	Obs:6/4 Exp: 4.9/5.1	Obs:76/56 Exp: 64.7/67.3	Obs:0/2 Exp:0.98/1.02	Obs:22/46 Exp: 33.3/34.7	13.8	0.008

Obs, observed; Exp, expected; *χ*^2^, chi-square; *p*, *p*-value.

There were no significant differences in age distribution in any of the direct comparisons; however, we repeated the analyses after excluding 29 individuals who were older than 26 years and could, thus, be identified as statistical outliers. The results were overall confirmed, with marginal changes in the *χ*^2^ and *p* values (Tables 1–5 and Supplementary Tables S1, S2).

In addition, in this cohort, there was a significant difference for Q21. Specifically, families seem to produce a larger amount of undifferentiated and organic waste, while respondents living in non-family households produced larger amounts of plastic and metal waste (Supplementary Table S2).

Finally, to better understand the factors influencing FW-related behaviors, we generated an arbitrary score, assigning from 0 to 3 points for responses to questions Q8-19, as summarized in Supplementary Table S3. Score values were significantly higher for female students (*p* < 0.001 by Student’s *t*-test). In addition, students living in a non-family-type household as well as students living in a new household showed higher score values as compared, respectively, with those living with their families and with those living in an old household situation (*p* < 0.001 by Student’s *t*-test). The only factors significantly associated with score values in a linear regression analysis were gender and grocery shopping habits. The two factors showed moderate collinearity (VIF: 1.01–1.00); we therefore performed a multiple regression analysis and observed that grocery shopping habits were the most relevant factor, explaining 45% of the score variance, with gender adding a further 2.6%.

## 4 Discussion

In this preliminary, descriptive study, we investigated FW-related habits of students enrolled at the University of Catanzaro Magna Graecia in Calabria, a Southern Italian region. Overall, the results of our survey show a high prevalence of virtuous behaviors in the food purchasing phase, with almost 90% of the entire group regularly checking for expiration dates, at least when buying perishable food items, and ~70% of them following a written or mental list/menu, when grocery shopping. In contrast, at home, less than 50% of respondents apply easy-to-implement waste prevention rules, such as storing purchased food according to its expiration date or recording expiration date when removing items from their original packaging. Misplanned food purchases and inadequate food storage have been described as key determinants of FW (23); however, in our cohort, we were unable to observe significant differences in typology (Q23) or frequency (Q22) of FW according to these characteristics. It is worth noting that, since several studies have demonstrated that consumers are unable to correctly estimate the amount of waste produced in their household, especially when filling out online questionnaires (23–26) we did not include quantitative questions in our survey. Qualitatively, the most common type of FW was spoiled fruits and vegetables, which is consistent with previous literature data (10, 23, 24, 27). Meal leftovers and expired, unused products shared the second position, while, contrary to previous reports (10, 25, 27–29) stale bread was the most frequent food discarded by a comparatively low percentage (7.2%) of respondents. This is all the more remarkable, given that, in our survey, bread and fruits/vegetables were the only categories specifically mentioned as FW typology, while all other food items were grouped into the two main categories of “meal leftovers” and “expired unused products”; thus, it would have been possible to anticipate a higher number of respondents choosing “bread” as an answer. It may be possible to hypothesize that the symbolic religious value of bread is still stronger in the Calabrian region (30) than in less traditionally oriented regions, even if the cohort recently analyzed by Fanelli et al. (28) as well as the students interviewed by Mondéjar-Jiménez (10) lived in Southern and Central Italian Regions that share a similar cultural heritage with Calabria.

Interestingly, more than 90% of the whole cohort declared following the rules for separate waste collection, and only 2.3% stated that a collection system has not been implemented in their area of residence. The presence of an organized and efficient separate waste collection organization has been suggested to be associated with a reduction of FW, as it likely increases environmental sensitivity and heightens the care devoted to waste disposal (13, 24). As far as waste typology is concerned, the prevalence of respondents who indicated “plastic and metal” as the most common waste appears also noteworthy. Single-use packaging is, by far, the major application of plastic in Europe (40%) (31) and, even if feasible, at home reusing of plastic containers (such as plastic bottles refilled with other beverages, especially hot ones) is not recommended as it has been demonstrated that reused plastic releases an increased amount of ftalates (32). The burden of reducing plastic waste thus lays on producers rather than consumers, but it has been proposed that the two issues would benefit from being addressed together as there are significant overlaps in possible optimizing strategies (33).

In addition, we observed several significant differences when comparing subgroups based on established or putative determinants of FW behaviors (gender, housing situation, degree program); however, none of these differences survived matching for group size and relevant habits (Section B questions). The only exception was the more appropriate handling of “use by” products by respondents who received structured nutrition teaching. We believe that this observation reinforces the importance of adequate education in the nutrition field (34).

Overall, direct involvement in grocery shopping appears to be the major determinant of our arbitrarily generated food wasting score. This observation is contrary to previous data suggesting that younger consumers have a lower ability to manage household food consumption (35); it could, however, be interpreted as a sign of the increasing attention toward environmental issues in the younger generations, who, when living independently, may choose to adopt more virtuous behaviors than those of their family of origins.

Interestingly, the only other variable giving a contribution, albeit minimal, to explain the score was gender. Gender has, indeed, been previously shown to influence FW related behavior. Women have been reported to have better knowledge of leftover processing and, particularly mothers of young children, tend to consume meal leftovers themselves rather than throwing them away (36, 37). However, they may also be more inclined than men to discard leftovers in their attempt to provide healthy and fresh meals to their family (38) and a decade-old Finnish study surprisingly shows increased FW rates in households where a woman was primarily in charge of grocery shopping (39). Also, girls seem to waste more food than boys in the school lunchrooms, and this appears to be due to their desire to eat healthier food (16). The challenges associated with conjugating high nutritional quality with low environmental impact have been highlighted also by a recent study in a French student cohort, where the Authors observed that independently living students tend to be more environmentally aware, in keeping with our observations, while students living with their family of origin followed a healthier diet. Interestingly, they did not observe any gender-related association; this may be partly explained by the socio-cultural differences between French and Italy (17). Indeed, unadjusted comparisons between the two genders show that women in our cohort are more frequently in charge of menial tasks, such as storing food items or cooking for the whole household, while males more frequently cook special meals. These observations highlight the need to better understand the drivers of FW and further underline the importance of an adequate education in the nutrition field, encompassing different aspects from health eating to FW reduction.

Our choice to carry out the survey by online questionnaires has pros and cons. First of all, the participation was voluntary; this may result, as previously shown, in a selection bias toward more aware and interested individuals; however, students were invited to participate during regular frontal lessons on subjects related as well as unrelated to nutrition issues and did not know the survey content in advance. The choice to omit from the formal analysis the respondents who did not correctly follow the survey instructions was, in fact, aimed at obtaining more robust results, since it can be hypothesized that these respondents had not paid enough attention while filling out the online form. It has also been observed that online anonymous questionnaires reduce the social desirability bias; to this end, we also employed a neutral tone and supplied “face-saving” alternatives whenever appropriate to encourage honest answers (40).

As noted above, quantitative data have not been included in our questionnaire, as it has been demonstrated that they cannot be reliably obtained through online forms (24, 25, 41). We also favored short straightforward questions to increase the number of respondents filling out the whole questionnaire; we were therefore unable to gather information on a number of factors which have been suggested, in previous studies, to affect FW related behaviors, including preferred shopping venues (supermarkets vs. local stores etc.), number and age of individuals sharing the same household, household income, frequency of not eating at home. The low mean age of our cohorts makes possible to infer that respondents living with their own new family will have young children, and the number of individuals under the age of 18 in a household has been reported to be associated either with a higher (42) or with a lower (27) rate of FW; however, the number of students (*n* = 13) living with their new families was too low to allow detecting any significant difference. By contrast, it has been suggested that individuals older than 65 have a greater awareness toward FW (21, 43) even if some studies do not confirm this observation (25, 44), parents of university age individuals, as those enrolled in our study, belong, likely, to a lower age range; thus, obtaining information on the household composition, including the presence of grandparents and older adults, could probably have helped to address this aspect. We also did not ask information on household income, which has been shown to represent a key determinant of FW (29); nonetheless, we hypothesize that the highest score of respondents living in a new housing situation as compared to those living with their family of origins may likely be in part determined by the lower economic possibility of younger families and students living on their own.

In conclusion, our results highlight the potential benefits of extending nutrition education to all university programs to allow young adults to acquire appropriate knowledge, which may help them in their quest to reduce FW preserving at the same time their health as well as the health of the planet (34).

## Data availability statement

The raw data supporting the conclusions of this article will be made available by the authors, without undue reservation.

## Ethics statement

The requirement of ethical approval was waived by Comitato Etico Azienda Ospedaliera “Mater Domini” for the studies involving humans because answers were anonymous and non-sensitive data only were collected. The studies were conducted in accordance with the local legislation and institutional requirements. The participants provided their written informed consent to participate in this study.

## Author contributions

FC: Formal analysis, Investigation, Writing – original draft, Writing – review & editing. VC: Investigation, Writing – original draft. AP: Conceptualization, Writing – review & editing. AS: Writing – review & editing. MH: Conceptualization, Formal analysis, Investigation, Writing – original draft, Writing – review & editing.
