# Supplementary material for: Food waste awareness among Italian university students: results of an online survey
Source: Front Nutr. 2024 Aug 5;11:1401581. doi: 10.3389/fnut.2024.1401581 (PMC11333036; doi:10.3389/fnut.2024.1401581)
Supplement: Supplementary file 1 [file Image_2.pdf]

Questionnaire

Section A

Q1. Age (optional)

Q2. Gender (optional)

Q3. What academic program are you currently attending?

A. Sport Science
B. Dietistics
C. Nursing
D. Psychology (Bachelor or Master course)
E. Pharmacy
F. Biotechnology (Bachelor)
G. Biotechnology (Master)
H. Other health care professions (no nursing or dietistics)
I. Bioengineering (Bachelor or Master course)
L. Law School
M. School of Economics and Management
N. Medicine
O. Dentistry

Q4. What is your living situation?

A. I live alone
B. I live with my family of origin (siblings, parents, grandparents)
C. I live with a partner or spouse
D. I live with my family (partner/spouse, children)
E. I live with roommates (less than 3)
F. I live with roommates (more than 3)
G. I live in a community (student dormitory, college, etc.)

Section B

Q5. Do you usually do your own grocery shopping?

A. Yes, always
B. Yes, usually, taking turns with the people who live with me.
C. Sometimes (less than once a month)
D. Rarely, only when no one else has the opportunity and I buy only essential items
E. No, never

Q6. Do you personally store food items in the cupboard/refrigerator/freezer?

A. Yes, always
B. Yes, every time I personally go grocery shopping.
C. No, even when I personally do the shopping, I only store the products that need to be refrigerated in the refrigerator/freezer and leave the other items to be properly stored by someone else.
D. No, even if I do the shopping, someone else stores the food items when I get home.
E. No, I never go the grocery shopping and I never take care of storing food items

Q7. Do you regularly cook your own meals?

A. Yes, usually, for myself

B. Yes, I usually cook for myself and for my household
C. Sometimes, in our household we take turns cooking.
D. No, I almost never cook, if I'm home alone I eat cold food or buy ready-made food
E. Only for special occasions
F. Open answer

Section C

If you answered D or E to Q5 go directly to Section D (Q12)

Q8. Do you use a shopping list when you go to the supermarket/grocery stores?

A. Yes, a written one
B. I don't prepare a written list, but I mentally plan the week menu when I go shopping
C. I don't prepare a list, sometimes I write down important items that I might otherwise forget
D. No, I buy what catches my attention
E. No, I usually buy the usual products that I know will be consumed

Q9. Do you look at the expiration date when you buy food products?

A. Yes, always
B. Only for perishable products
C. Rarely

Q10. Do you know the difference between "use by" and "best before"?

A. Yes, I do
B. Yes, I think so
C. No, I have no idea
D. There isn't any difference

Q11. Do you keep a regular schedule for grocery shopping?

A. No, I go when I can, according to other commitments
B. Yes, I usually go on weekdays, in the morning
C. Yes, I usually go on weekdays, at lunchtime
D. Yes, I usually go on weekdays, in the evening
E. Yes, I usually go on the weekends, in the morning
F. Yes, I usually go on the weekends, at lunchtime
G. Yes, I usually go on the weekends, in the evening

Section D

If you answered C, D or E to Q6 go directly to Section E (Q16)

Q12. Do you follow a criterion when storing food items in the cupboard (products that do not need refrigeration)?

A. Yes, I divide them by type, and I put in the back the newly purchased items
B. Yes, I store the items in order of expiration date (those that expire before in the front)
C. Yes, according to package size
D. No, I just try to optimize the available space.
E. No

Q13. Do you follow a criterion when storing food items in the refrigerator?

A. Yes, I store the items in order of expiration date (those that expire earlier in the front)
B. No, I just try to optimize the available space.
C. Yes, I divide them by type, and I put in the back the newly purchased items

D. No
E. Open answer

Q14. Do you follow a criterion when storing food items in the freezer?
A. Yes, I store in the front the products with the earlier expiration date
B. No

Q15. If you remove food items from their packaging to store them (e.g., eggs), do you record the expiration date?
A. Yes, on a wall calendar that I keep in the kitchen
B. Yes, on my cell phone or on a personal calendar
C. No, but I plan my purchases based on consumption so they don't expire
D. No, I kind of remember the date of purchase and I roughly calculate the expiration date accordingly
E. No, I evaluate whether the products are still good before eating them (e.g., if an egg floats in a glass of water, I throw it away)
F. I never take products off their packaging
G. Open answer

Section E
If you answered D or E to Q6 go directly to Section F (Q20)

Q16. What do you do with fruits and vegetables that are no longer fresh?
A. I throw them away
B. I try to use the parts that are still good
C. I cook them so that they still have a pleasant taste and can be consumed

Q17. What do you do with meals leftovers?
A. I throw them away
B. I eat them at later meals
C. I very rarely have leftovers, I pay a lot of attention to the quantities when preparing meals

Q18. If a product with a "use by" label is 1 week past its expiration date, what do you do?
A. If the taste is not altered, I use it to prepare meals
B. I use it to prepare meals, discarding any mold or too damaged portions
C. I throw it away, it's dangerous to consume it
D. I use it to prepare meals only if they will be consumed exclusively by adults and healthy people
E. It hardly ever happens, I plan my purchases and consumption well and do not let food go past its expiration date

Q19. If a product with a "best before" label is 1 week past its expiration date, what do you do?
A. If the taste is not altered, I use it to prepare meals
B. I throw it away, it's dangerous to consume it
C. I use it to prepare meals, it is unlikely that it is no longer consumable
D. It hardly ever happens, I plan my purchases and consumption well and do not let food go past its expiration date

Section F

Q20. Do you follow separate waste collection regulations?

A. Yes, I do
B. No, there no specific rules in the area where I live
C. I try, but I frequently make mistakes
D. No, I find them boring and useless

Q21. If you follow separate waste collection regulations (or try to), which kind of waste do you produce in greater amounts?

A. Undifferentiated/residual dry
B. Plastic/Metal
C. Paper
D. Glass
E. Organic/compostable
F. I don't know

Q22. How often do you (or your household) throw away food leftovers?

A. Almost every day
B. More or less once a week
C. A couple of times a month
D. Once a month or less
E. It's a very rare event

Q23. Which of the following is the most common food waste in your household?

A. Fruit or vegetables, gone bad
B. Expired products, not consumed
C. Meal leftovers
D. Stale bread
E. I throw away all products quite frequently
F. I almost never throw anything away
